# Supplementary material for: Improving Social Justice in COVID-19 Health Research: Interim Guidelines for Reporting Health Equity in Observational Studies
Source: Int J Environ Res Public Health. 2021 Sep 4;18(17):9357. doi: 10.3390/ijerph18179357 (PMC8431098; doi:10.3390/ijerph18179357)
Supplement: Supplementary file 1 [file ijerph-18-09357-s001.zip › ijerph-1337348-supplementary.pdf]

Supplementary Material S1

Contents

Table S1. Checklist of possible items for STROBE- Equity reporting guidelines	2
Additional details of equity in COVID-19 observational studies	7
Table S2. Additional examples of COVID-19 observational studies	12
References	15

Table S1. Checklist of possible items for STROBE- Equity reporting guidelines

	Item No	Standard STROBE checklist	Possible items for STROBE- Equity extension
**Title and abstract**	1	1a. Indicate the study's design with a commonly used term in the title or the abstract<br><br>1b. Provide in the abstract an informative and balanced summary of what was done and what was found	Describe population according to PROGRESS-Plus<br><br>Describe extent/limits of applicability to populations of interest across PROGRESS-Plus characteristics
**Background, rationale**	2	Explain the scientific background and rationale for the investigation being reported	If health equity is a focus, what is the rationale for focus on health equity, across PROGRESS-Plus (if relevant)?
**Objectives**	3	State specific objectives, including any pre specified hypotheses	
**Study design**	4	Present key elements of study design early in the paper	Report who was involved, engaged or consulted in study design (e.g., community, industry, government, etc.)
**Setting**	5	Describe the setting, locations, and relevant dates, including periods of recruitment, exposure, follow-up, and data collection	Report whether methods of sampling/recruitment were designed to reach populations across relevant PROGRESS-Plus characteristics<br><br>Report possibility of self-selection bias across PROGRESS-Plus factors
**Participants**	6		
		6a. *Cohort study*—Give the eligibility criteria, and the sources and methods of selection of participants. Describe methods of follow-up<br><br>*Case-control study*—Give the eligibility criteria, and the sources and methods of case ascertainment and control selection. Give the rationale for the choice of cases and controls<br><br>*Cross-sectional study*—Give the eligibility criteria, and the sources and methods of selection of participants	Give inclusion and exclusion criteria across relevant PROGRESS-Plus characteristics<br><br>Report context and relationship to health equity (additional items may be needed to document context and systems in which the studies take place)<br><br>Report details of partnerships with populations and communities, where applicable

		6b. *Cohort study*—For matched studies, give matching criteria and number of exposed and unexposed<br><br>*Case-control study*—For matched studies, give matching criteria and the number of controls per case	Report whether any PROGRESS-Plus factors used for matching, how categories were determined and why
**Variables**	7	Clearly define all outcomes, exposures, predictors, potential confounders, and effect modifiers. Give diagnostic criteria, if applicable	Report whether outcomes were identified as relevant and important to populations across PROGRESS-Plus
**Data sources, measurement**	8	* For each variable of interest, give sources of data and details of methods of assessment (measurement). Describe comparability of assessment methods if there is more than one group	Report the method of obtaining population characteristics (e.g., age)
**Bias**	9	Describe any efforts to address potential sources of bias	Report efforts to reduce selection bias across PROGRESS-Plus<br><br>Report whether dimensions of context might influence the study (e.g., bias in response/participation)
**Study size**	10	Explain how the study size was arrived at	Report whether PROGRESS-Plus characteristics of interest were considered in determining the study size
**Quantitative variables**	11	Explain how quantitative variables were handled in the analyses. If applicable, describe which groupings were chosen and why	Report how decisions were made about analyses related to PROGRESS-Plus, including whether any categories were defined, and how they were decided<br><br>Report whether dimensions of context were collected for analysis
**Ethical concerns**			Report details of informed consent and ethical clearance, particularly for populations vulnerable in the context of research
		12a. Describe all statistical methods, including those used to control for confounding	If PROGRESS-Plus factors used to control for confounding, describe how they were defined and rationale<br><br>Report whether contextual factors were used in adjustment for confounding

		12b. Describe any methods used to examine subgroups and interactions	Report details of additional analyses related to health equity<br><br>Report whether context or systems were explored
		12c. Explain how missing data were addressed	Explain whether missing data was related to individual or contextual factors associated with health inequities
		12d. *Cohort study*—If applicable, explain how loss to follow-up was addressed<br><br>*Case-control study*—If applicable, explain how matching of cases and controls was addressed<br><br>*Cross-sectional study*—If applicable, describe analytical methods taking account of sampling strategy	
		12e. Describe any sensitivity analyses	
**Participants**	13	13a.* Report numbers of individuals at each stage of study—e.g. numbers potentially eligible, examined for eligibility, confirmed eligible, included in the study, completing follow-up, and analysed	
		13b.* Give reasons for non-participation at each stage	Describe the losses and exclusions of participants across PROGRESS-Plus<br><br>Describe non-response/non-participation across PROGRESS-Plus
		13c.* Consider use of a flow diagram	
**Descriptive data**	14	14a.* Give characteristics of study participants (e.g. demographic, clinical, social) and information on exposures and potential confounders	Present characteristics across relevant PROGRESS-Plus characteristics
		14b.* Indicate number of participants with missing data for each variable of interest	Describe whether data on PROGRESS+ factors are missing (e.g. ethnicity data in some settings has a high level of missing-ness)
		14c.* *Cohort study*—Summaries follow-up time (e.g., average and total amount)	

**Outcome data**	15	*Cohort study*—Report numbers of outcome events or summary measures over time<br><br>*Case-control study*—Report numbers in each exposure category, or summary measures of exposure<br><br>*Cross-sectional study*—Report numbers of outcome events or summary measures	
**Main results**		16a. Give unadjusted estimates and, if applicable, confounder-adjusted estimates and their precision (e.g., 95% confidence interval). Make clear which confounders were adjusted for and why they were included	Report if confounders were defined for contextual or PROGRESS+ factors that are associated with health inequities<br><br>Justify why certain categories of PROGRESS+ are not disaggregated for analysis
		16b. Report category boundaries when continuous variables were categorised	Justify any categories used across PROGRESS-Plus characteristics
		16c. If relevant, consider translating estimates of relative risk into absolute risk for a meaningful time period	
**Other analysis**	17	Report other analyses done (e.g., analyses of subgroups and interactions, and sensitivity analyses)	Report other analyses to address health equity questions, if the study had objectives related to health equity
**Key results**	18	Summarise key results with reference to study objectives	
**Limitations**	19	Discuss limitations of the study, taking into account sources of potential bias or imprecision. Discuss both direction and magnitude of any potential bias	
**Interpretation**	20	Give a cautious overall interpretation of results considering objectives, limitations, multiplicity of analyses, results from similar studies, and other relevant evidence	Consider importance of context in interpretation of health equity
**Generalizability**	21	Discuss the generalizability (external validity) of the study results	Discuss external validity to populations across relevant PROGRESS-Plus characteristics, considering issues of possible self-selection, healthy volunteer bias, losses across PROGRESS-Plus

			Consider implications of exclusion of people across PROGRESS as well as differential participation and/or loss to follow-up<br><br>Consider context in discussion of generalizability
**Funding**	22	Give the source of funding and the role of the funders for the present study and, if applicable, for the original study on which the present article is based	

Abbreviations: PROGRESS-Plus, Place of residence, Race/ethnicity/culture/language, Occupation, Gender or sex, Religion, Education, Socioeconomic status, Social capital, and other contextual factors that facilitate disadvantage.
*Give information separately for cases and controls in case-control studies and, if applicable, for exposed and unexposed groups in cohort and cross-sectional studies.

Additional details of equity in COVID-19 observational studies

Place of residence

Place of residence refers to the location where an individual or group lives and involves the availability of adequate infrastructure and allocation of services [1,2]. Place of residence has been commonly referred to in terms of a geographical area, such as country (e.g., high, middle, or low income) and also within countries, and can be broken down into further strata (e.g., neighbourhoods, dwelling type) [1,3,4]. Statistical techniques and methods including spatial clustering analysis and small-area analyses enabling overlay of data at different scales [5] have been used to investigate geographical patterns and clusters of COVID-19 risk, spread, and mortality at the population level. Geocoding also allows new opportunities for analyses of place-based information and socioeconomic gradient [6]. For example, to assess the geographic accessibility to oxygen-ready facilities for patients with COVID-19 [7]. While strong associations with COVID-19 infection risk and place of residence have been reported, analyses and interpretations of observational research are further complicated as residence is linked to related factors such as occupation (or place of work) which may present additional interactions for risk and level of exposure. Individual-level data, if available, may include the participant's address, postal/zip code, city, or type of dwelling. Researchers should be aware of the ecological fallacy for interpretations of relationships at the individual level based on aggregate-level data [5]. For example, when collecting place of residence metrics, 'pockets' of inequity may be missed (e.g., inner city, shelters), and researchers may consider adjusting analyses by centralised marginalisation indices where possible.

- An individual's risk for COVID-19 infection has been closely associated with risks due to spatial factors such as housing and congregate living facilities [4,5]. Congregate settings including assisted living, long-term care and retirement homes, many of which have had outbreaks over the course of the pandemic. However, there has been limited epidemiological research, partly due to varying models of organization [6] in these settings. Many factors, such as negligence, poor planning and allocation of resources (including access to protective equipment and healthcare workers), can be characterised as avoidable and therefore inequitable.
- Postal and zip codes have been used in epidemiological studies of COVID-19 to identify 'hotspots' for disease burden. For example, individuals living in rural communities face greater barriers to healthcare access (e.g., fewer healthcare workers available, absence of facilities) [1]. It has been highlighted that COVID-19 transmission data in rural communities is lacking [7], hampering public health efforts to control its spread in these regions.
- Based on spatial analyses of population-level US data, areas with dense populations, airports, and higher levels of air pollution conferred higher risk for COVID-19 related mortality [8]
- Missed opportunities to account for differences attributable to place of residence and other factors can result in biased estimates (i.e., exposure misclassification) [4] and impact the internal and external validity of the study. For example, individuals living in rural settings that travel to urban centres with higher COVID-19 prevalence for work [8] or to collect government compensation payments [9].

Race, ethnicity, culture or language

Race assigns people to socially constructed categories based on phenotypes, while ethnicity comprises sociocultural characteristics (e.g., language and culture) and characteristics of relationships between ethnically defined groups and the society within which they exist [8]. Race and ethnicity are time- and context-specific constructs with multi-level implications (i.e., personal identity at individual and group-level) that are not necessarily based on natural differences [8,9]. The COVID-19 pandemic has exacerbated health inequities across racial and ethnic groups. For example, a systematic review found that Black populations have one and a half to three-fold risk of infection and hospitalisation compared with White populations [10]. Therefore, to investigate COVID-19 disparities, a strong understanding of racial and ethnic stratification is necessary [11]. Further, race-based and ethnicity data

collection should be conducted carefully and ideally in partnership with the community being studied [12].

- Ethnic minorities are at a higher risk of getting infected and dying from COVID-19 [10].
- Due to social constructs, ethnic minorities are more likely to live in areas with higher incidence of COVID-19 [11].
- Ethnic minorities are disproportionately represented in health care facilities, food and retail services among other essential work settings [12].
- Ethnic groups tend to have different levels of knowledge and attitudes concerning COVID-19 disease progression, personal prevention practices and management [13,14].

Occupation

Occupation implies different situations in which a person experiences vulnerability as a direct result of their place of work or employment status (i.e., employed, unemployed, or retired). Occupational hazards have long been known to result in disparities in health (e.g., asbestos exposure) [13] and these disparities have been exacerbated during the COVID-19 pandemic [14]. Specifically, many groups of workers were considered essential to the well-being of the community, which resulted in a systematic increased risk of disease solely based on the individual's occupation [14]. Importantly, essential workers also experienced differential vulnerabilities across other PROGRESS-Plus factors such as ethnicity [15], socioeconomic status, and gender (e.g., in the European Union, [16]and this resulted in further health disparity.

- Healthcare workers and care providers are directly impacted by the pandemic and are at higher risk of disease than peers who can maintain social distancing and isolation practices [17].
- Many who work manual labour jobs (e.g., meat packing, farm labour) were already at risk [18] and outbreaks in seasonal and migrant workers were seen worldwide [19,20].

Gender and sex

Gender refers to socially constructed roles, responsibilities, attributes, and entitlements to be (or being perceived as) a woman, man, or gender diverse individual in a particular setting, along with the distribution of power between and among groups by gender [20,21]. Sex refers to a set of biological traits that usually distinguishes females, males, and individuals with differences in sex development (i.e., variations in chromosomal expressions or physiological characteristics that differ from the female-male dichotomy) [20,22]. Biological characteristics may have a role in COVID-19 related mortality (higher among male individuals) and long-term effects of COVID-19 (higher among female survivors) [23,24], while health inequities mainly reflect stratifying forces with impacts on gender [21]. For example, in Canada, Indigenous women and girls are more likely to experience violence than other groups [25]. Currently few countries provide sex- and gender-disaggregated data on COVID-19 data [26]. Also COVID-19 observational studies rarely reported sex-disaggregated main outcomes, analysed sex- and gender-based differences, or discussed implications across sex and gender [27,28].

- It has been hypothesised that COVID health outcomes are associated with either biological susceptibility (e.g., stronger immune response in females) or gender-related behaviours (e.g., higher likelihood of smoking and drinking among men) [21–23].
- Severe adverse effects following COVID-19 vaccination occurred more frequently in female participants [sex] [24].
- Pregnant females [sex] with suspected or confirmed COVID-19 are at increased risk of admission to an intensive care unit compared with non-pregnant females of reproductive age [25]. Gendered differences in exposure are related to other intersecting factors, for example, occupation risk in a gendered distribution of work, wherein women are highly represented in essential occupations during the COVID-19 outbreak, including the health workforce [26,29,30].
- Gender norms exacerbate barriers to healthcare systems, for example, men can be more reluctant to seek care, and women can lose autonomy in decision-making [27,28].
- Gender diverse people can experience greater challenges regarding their mental health and those who are undergoing transition-related treatment can face accessibility constraints [83].

- Pandemic policies and public health measures have different implications by gender. For instance, during lockdowns, violence against women has intensified [31–33], and the gap in the distribution of unpaid care work has increased [33].

Religion

Religion implies a complex interweaving of relations between individual beliefs, social practices and norms, and community services that influence health outcomes [31]. Structural racism also promotes the discrimination of some faith communities. For example, in Europe, residential segregation is mainly driven by religion [33]. Religious beliefs and practices positively and negatively influence disease stigma, health-seeking behaviour, and preparedness of community and health systems [32,34]. Previous experiences with other infectious disease outbreaks highlight the challenges for implementing disease control measures [35,36]. The World Health Organization has recognised religious institutions as contributors to the promotion of healthy hygiene practices and to fear prevention during the pandemic. However, it has also expressed concern about religious practices that involve large gatherings and may increase community spread of COVID-19 [37]. Public health interventions may benefit from and/or unfairly exclude specific communities based on their religious convictions.

- Faith-based groups can be associated with different knowledge, perceptions, and attitudes toward COVID-19 [35–37].
- Public health measures and policy-makers need to consider the risk for community transmission of large religious congregations [38].
- Religious leaders and faith-inspired communities represent potential public health stakeholders to enhance adherence to mitigation policies [39].

Education

Education refers to an individual's level of formal educational attainment (e.g., high school, college, university), or measured as years of education. Conceptually, education can extend beyond skills attained in 'formal' school settings [38]. Education is associated with better health and it has been well documented that people with higher levels of education are more likely to follow a healthy lifestyle (i.e., protective behaviour), practice preventative health, and to have better capacity for self-management [1,38]. In the COVID-19 context, higher levels of education are associated positively with vaccine acceptance [40]. Data for education is often readily available for research purposes [41], and its collection (and any operationalization of it) should be clearly outlined.

- Misinformation, about transmission and treatment for COVID-19, has presented major challenges to public health efforts. Cross-sectional studies have reported on factors associated with greater knowledge about COVID-19 including greater than high school or 'qualified to attend university' levels of education [41,42], having educated parents, and obtaining health information from sources such health professionals as opposed to social media [37], which was also strongly associated with preventive behaviours.
- Individuals who have higher levels of education are less likely to smoke [43–45]. Smoking has been associated with increased risk for respiratory diseases including COVID-19, although large observational studies exploring associations with severity of COVID-19 symptoms and intensive care unit admissions have been very limited [46,47].
- Cross-sectional studies evaluating vaccine acceptance have reported an association with education. Fewer individuals preferred domestic vaccines over vaccines from abroad when the level of education increased [48], highlighting important challenges for uptake. Higher levels of education and the perceived trustworthiness of sources for COVID-19 information (healthcare providers and disease-specific societies) were associated with greater vaccine willingness [49].
- Researchers should be cautious when making any assumptions regarding health literacy and education, since this may also be related to culture and language [50].

Socioeconomic status

Socioeconomic status (SES) is traditionally defined by an individual's occupation, income, and education [42]. When operationalizing SES in research, economic indicators were found to capture as much variance in health outcomes compared to other indicators [43,44]. Disparities in SES account for the most fundamental cause of health inequities in the COVID-19 pandemic and are encompassed by three major determinants: health care access, environmental exposure, and health behaviour [45]. These determinants interact to influence the pervasive effect of health inequities. The underfunding of public health and undermining of equitable health care access has resulted in poor access to high quality primary and speciality care within marginalised communities [46]. In parallel, COVID-19 has disproportionately impacted low-income communities, with infection rates six or seven times higher than those of higher-income communities [51]. The socioeconomic gradient is also associated with self-protective behaviours during the COVID-19 pandemic (e.g., lower SES is associated with reduced ability to distancing physically due to more crowded housing) [47] The variation in population health across SES needs to be investigated to develop effective policies that can reduce the harmful effects of disparities in income and access [48].

- The COVID-19 pandemic differently impacts the mental and physical health of individuals with low SES due to unstable work conditions and unstable income [52,53].
- Individuals with low income tend to work in essential worker jobs such as retail and food services, and therefore, are at a higher risk of infection and mortality [54].
- Individuals with lower education and income practiced different risk-adverse behaviours such as vaccination and wearing masks from those with higher education [55].
- Economically disadvantaged individuals have a greater likelihood of living in overcrowded accommodations [56].
- Low SES is strongly associated with comorbidities which increases the vulnerability to COVID-19 [57,58].
- Individuals with low SES tend to access health care at more progressive stages of illness which would likely lead to poorer health outcomes [59].

Social capital

Social capital can be described as the health resources derived from social connections that encompass trust between individuals and standards of reciprocity [49] and can be gained as a result of other factors (e.g., occupation, income). The impact of social capital can be particularly visible in the extremes - those who have much capital and those who have little. In practice, social capital may enable preferential treatment in some (e.g., early access to therapy for an influential or public figure) and remove protections from another (e.g., inmates exposed to disease within a correctional facility [52]. The types of social capital include bonding (i.e., close relationships), bridging (i.e., more distant relationships, such as neighbours), and linking social capital (i.e., relationships that are defined by a power asymmetry, such as between employer and employee) [53]. Social capital may play either a buffering role against poverty effects on health or a dependent behaviour on SES, depending on the circumstances (e.g., SES thresholds allow accumulating particular types of social capital) [53].

- Inmates are often considered of low social status and significant outbreaks were observed within correctional facilities [60].
- Refugees, migrants, and homeless populations were heavily impacted directly by the pandemic and indirectly by the national response (e.g., border and service closures) [61–64].
- The impact of social lockdowns was noted to disproportionately reduce the risk of disease by social standing in France [65].
- The uptake of misinformation and negative attitudes toward public health measures were both localized and aligned by voting blocks in the USA, suggesting that these attitudes were held to gain or retain social capital within personal networks [66,67].

Plus

The Plus in PROGRESS-Plus refers to context-relevant personal characteristics (e.g., age, disability), features of relationships, and time-dependent relationships (e.g., people in prisons) that drive variations in health.

Other Social Groups

Impacts of public health responses have differentially affected other social groups in their opportunities for health, such as children (e.g., school meal scheme disruption), people with disabilities (e.g., reduced access to routine rehabilitation), refugee and displaced populations (e.g., overcrowded living arrangements, vaccine access) [56], people experiencing homelessness (e.g., possibilities to follow infection control measures), and Indigenous communities [58].

Indigenous Peoples

Intersectionality plays an important role in Indigenous health inequities. The historical context of colonization experienced by Indigenous Peoples requires consideration in analyses. Acts of genocide by colonial states (e.g., residential schools, forced removal of children from families, forced sterilization, etc.) have significant impacts on the health and wellbeing of communities, families and individuals [57]. COVID-19 amplifies the risk of inequities in health experienced by Indigenous Peoples [59]. Inequitable healthcare provision continues in the present day. For example, during the H1N1 pandemic, body bags were sent to First Nations communities in need of assistance [61]. In the Canadian context, health research with respect to First Nations, Inuit and Métis Peoples has been grounded in a history of colonization and paternalism which has lacked appropriate collaboration with Indigenous Peoples [62,63]. Historically, western-oriented research approaches have failed to engage Indigenous communities in the research process. A societal change is needed to ensure that reporting results of such examinations include Indigenous perspectives [64–66]. Critical examination of structural inequities and policy decisions regarding Indigenous Peoples is required to enable an informative and participatory decision-making process (e.g., using available epidemiological data to prioritise vaccination) [68].

- People with disabilities and elderly people living in congregate settings are at higher risk of infection and illness severity because of exposure (e.g., physical proximity during personal care) and comorbidities [40,68].
- Difficulties in accessing healthcare and their concerns and needs have been taken into consideration by healthcare professionals and policymakers [68].
- The consequences of school closures are more severe among vulnerable children. These impacts go beyond interrupting learning, and compromise poor nutrition and increase risks to violence and exploitation [69,70].
- Indigenous communities suffer disproportionately from the effects of COVID-19 because of historical oppression and violence, including under-resourced health services and lack of consideration for their unique beliefs, culture, and traditions [71]. Thus, public health measures, such as vaccination campaigns, need to consider particular challenges and opportunities [72].

Multimorbidity

Multimorbidity refers to the coexistence of multiple chronic conditions that additively place patients at a greater risk of worse health outcomes [69]. Multimorbidity intersects extensively with other social determinants of health, such as place of residence, socioeconomic status, and social capital, thereby exacerbating the poor outcomes experienced by such patients [69,70]. Recent studies have found several chronic diseases (i.e., diabetes, cardiovascular diseases, and chronic kidney disease) as prognostic factors for the severity of COVID-19 [71]. Observational studies that adjust for the presence of comorbid conditions provide evidence about whether differential health outcomes are associated with exposure-related factors and healthcare access or individual susceptibility[10].

Table S2. Additional examples of COVID-19 observational studies

PROGRESS-Plus factors	Level of analysis	Context<br>*Country*<br>*Data Source*	Gap or Focus approaches*	Topic	Differences in health opportunities	Research question	Study design
**Place of residence**	Group	44 Sub-Saharan African countries<br>Data sources for geolocation of health-care facilities	Gap	Healthcare access	Differential accessibility to healthcare facilities	What is the travel time to the nearest hospital and health-care facility for 60+ adults? [73]	Cross-sectional
	Individual	United States<br>City-level<br>Hospital health records	Focus	Prognosis	Differential exposure and susceptibility	What is the impact of densely populated residential areas on hospitalizations? Which are predictors of hospital stay and mortality? [74]	Cohort
**Race, ethnicity, culture, or language**	Group	United States<br>County-level data	Focus	Prognosis	Differential accessibility to testing and healthcare	Is the proportion of non-Hispanic black persons in a US county an independent measure of population-level race disparities in COVID-19? [75]	Ecological
	Individual	United States<br>Hospital health records	Gap	Incidence	Differential exposure and access to healthcare	Are there differences in the hospitalisation an clinical outcomes between Black, White, and Hispanic White (Hispanic) COVID-19 hospitalised patients who present to the emergency department? [76]	Cohort
	Group	England<br>Local authority level data	Gap	Incidence	Differential exposure	Which is the relationship between ethnic composition of an area and rate of mortality i[illegible] the area? [77]	Ecological
**Occupation**	Group	England<br>Linked biobank and public health database	Gap	Incidence	Differential exposure	What is the risk of severe COVID-19 infection by occupational groups? [14]	Prospective cohort

**Gender**	Individual	Kenya, Nigeria, and South Africa	Gap	Healthcare access	Differential accessibility to health care	Are there differences in the impact on demand for and access to health care across genders? [78]	Cross-sectional
**Sex**	Individual	United States Hospital health records	Gap	Prognosis	Differential susceptibility	What are the individual risk factors, including sex, associated with intubation in hospitalised patients with COVID-19? [79]	Retrospective cohort
**Religion**	Individual	Australia Community-based	Gap	Knowledge, attitudes, perceptions	Differential acceptability	What are demographic, attitudinal, political and social attitudes, including religiosity, and COVID-19 health behaviour associated with vaccine hesitancy and resistance? [80]	Cross-sectional
**Education**	Individual	Brazil City-level blood donor database	Gap	Prevalence	Differential exposure	What is the seroprevalence of SARS-CoV-2 antibodies in asymptomatic blood donors? [81]	Cross-sectional
**Socioeconomic status**	Group	Australia Online survey University students	Focus	Prevalence	Differential exposure	Which is the level of psychological wellbeing among students during COVID-19? Which factors [including social status] are associated with lower wellbeing? [82]	Cross-sectional
	Individual	Mexico Ministry of Health database	Gap	Knowledge, attitudes, perceptions	Differential exposure and accessibility to healthcare	Are there differences in risk of hospitalisation with COVID-19 across sociodemographic, access to health care, and presentation to care characteristics [including municipality-level social deprivation index as a proxy of individual-level socioeconomic status] among individuals with diabetes? [83]	Cross-sectional
**Social capital**	Group	United States Population-based survey	Gap	Knowledge, attitudes, perceptions	Differential acceptability	What are the barriers to public health prevention measures? [84]	Cross-sectional
**Plus**	Individual Elderly	Spain Online survey	Focus	Policy impact	Differential response	Are older adults at higher psychological risk from COVID-19 pandemic and	Cross-sectional

people	lockdown? [85]

Abbreviations: SARS-CoV-2, severe acute respiratory syndrome coronavirus 2

* Definition provided by Welch and colleagues [86]; gap: study evaluates inequities between groups (e.g. genders), focus: study evaluates inequities only in a vulnerable group (e.g., essential workers).

References

1 O'Neill J, Tabish H, Welch V, *et al.* Applying an equity lens to interventions: using PROGRESS ensures consideration of socially stratifying factors to illuminate inequities in health. *Journal of clinical epidemiology* 2014;**67**:56–64. doi:10.1016/j.jclinepi.2013.08.005

2 World Health Organization. A guide to equitable water safety planning: Ensuring no one is left behind. 2019.https://www.who.int/publications/i/item/9789241515313 (accessed 15 Jun 2021).

3 Roux AVD. Investigating Neighborhood and Area Effects on Health. 2001.

4 Kirby RS, Delmelle E, Eberth JM. Advances in spatial epidemiology and geographic information systems. *Annals of Epidemiology* 2017;**27**:1–9. doi:10.1016/j.annepidem.2016.12.001

5 Dé F, Piel RB, Fecht D, *et al.* Small-area methods for investigation of environment and health. doi:10.1093/ije/dyaa006

6 Introduction | The Public Health Disparities Geocoding Project Monograph | Harvard T.H. Chan School of Public Health. https://www.hsph.harvard.edu/thegeocodingproject/introduction/ (accessed 7 Jul 2021).

7 Denhard L, Kaviany P, Chicumbe S, *et al.* How prepared is Mozambique to treat COVID-19 patients? A new approach for estimating oxygen service availability, oxygen treatment capacity, and population access to oxygen-ready treatment facilities. 2021;**20**:1–13.https://equityhealthj.biomedcentral.com/articles/10.1186/s12939-021-01403-8 (accessed 9 Jun 2021).

8 Ford CL, Harawa NT. A new conceptualization of ethnicity for social epidemiologic and health equity research. *Social Science and Medicine* 2010;**71**:251–8. doi:10.1016/j.socscimed.2010.04.008

9 Bhopal R, Donaldson L. White, European, Western, Caucasian, or what? Inappropriate labeling in research on race, ethnicity, and health. *American Journal of Public Health* 1998;**88**:1303–7. doi:10.2105/AJPH.88.9.1303

10 Mackey K, Ayers CK, Kondo KK, *et al.* Racial and Ethnic Disparities in COVID-19-Related Infections, Hospitalizations, and Deaths : A Systematic Review. *Annals of internal medicine* 2021;**174**:362–73. doi:10.7326/M20-6306

11 Pareek M, Bangash MN, Pareek N, *et al.* Ethnicity and COVID-19: an urgent public health research priority. The Lancet. 2020;**395**:1421–2. doi:10.1016/S0140-6736(20)30922-3

12 Funnell S, Tanuseputro P, Letendre A, *et al.* Nothing about us, without us. How community-based participatory research methods were adapted in an indigenous end-of-life study using previously collected data. *Canadian Journal on Aging* 2020;**39**:145–55. doi:10.1017/S0714980819000291

13 Repp K, Lorbeer R, Ittermann T, *et al.* Occupational exposure to asbestos is associated with increased mortality in men recruited for a population-based Study in Germany. *International Journal of Occupational Medicine and Environmental Health* 2015;**28**:849–62. doi:10.13075/ijomeh.1896.00549

14 Mutambudzi M, Niedwiedz C, Macdonald EB, *et al.* Occupation and risk of severe COVID-19: Prospective cohort study of 120 075 UK Biobank participants. *Occupational and Environmental Medicine* 2021;**78**:307–14. doi:10.1136/oemed-2020-106731

15 Rogers TN, Rogers CR, VanSant-Webb E, *et al.* Racial Disparities in COVID-19 Mortality Among Essential Workers in the United States. *World Medical & Health Policy* 2020;**12**:311–27. doi:10.1002/WMH3.358

16 European Insititute for Gender Equality. Essential workers. https://eige.europa.eu/covid-19-and-gender-equality/essential-workers (accessed 22 Jun 2021).

17 T S, H K, T P, *et al.* Disparities in rheumatoid arthritis disease activity according to gross domestic product in 25 countries in the QUEST-RA database. *Annals of the rheumatic diseases* 2009;**68**:1666–72. doi:10.1136/ARD.2009.109983

18 Upshaw TL, Brown C, Smith R, *et al.* Social determinants of COVID-19 incidence and outcomes: A rapid review. *PLoS ONE* 2021;**16**:e0248336. doi:10.1371/journal.pone.0248336

19 Castillo F, Mora AM, Kayser GL, *et al.* Environmental Health Threats to Latino Migrant Farmworkers. In: *Annual Review of Public Health*. Annual Reviews Inc. 2020. 257–76. doi:10.1146/annurev-publhealth-012420-105014

20 Coen S, Banister E E. What a Difference Sex and Gender Make: A Gender, Sex and Health Research Casebook - CIHR. Ottowa, Canada: Canadian Institutes of Health Research. 2012.https://cihr-irsc.gc.ca/e/44734.html (accessed 25 Feb 2020).

21 Heise L, Greene ME, Opper N, *et al.* Gender inequality and restrictive gender norms: framing the challenges to health. The Lancet. 2019;**393**:2440–54. doi:10.1016/S0140-6736(19)30652-X

22 Cools M, Nordenström A, Robeva R, *et al.* Caring for individuals with a difference of sex development (DSD): A Consensus Statement. *Nature Reviews Endocrinology* 2018;**14**:415–29. doi:10.1038/s41574-018-0010-8

23 Dehingia N, Raj A. Sex differences in COVID-19 case fatality: do we know enough? The Lancet Global Health. 2021;**9**:e14–5. doi:10.1016/S2214-109X(20)30464-2

24 Sudre CH, Murray B, Varsavsky T, *et al.* Attributes and predictors of long COVID. 2021;**27**:626–31. doi:10.1038/s41591-021-01292-y

25 Assembly of First Nations. Missing and Murdered Indigenous Women and Girls and Ending Violence - Assembly of First Nations - AFN. https://www.afn.ca/policy-sectors/mmiwg-end-violence/ (accessed 14 Jun 2021).

26 The COVID-19 Sex-Disaggregated Data Tracker | Global Health 50/50. https://globalhealth5050.org/the-sex-gender-and-covid-19-project/the-data-tracker/ (accessed 9 Jun 2021).

27 Palmer-Ross A, Ovseiko P v, Heidari S. Inadequate reporting of COVID-19 clinical studies: a renewed rationale for the Sex and Gender Equity in Research (SAGER) guidelines. *BMJ Global Health* 2021;**6**:e004997. doi:10.1136/bmjgh-2021-004997

28 Schiffer VMMM, Janssen EBNJ, van Bussel BCT, *et al.* The "sex gap" in COVID-19 trials: a scoping review. *EClinicalMedicine* 2020;**29–30**. doi:10.1016/j.eclinm.2020.100652

29 T P, R E, DA D, *et al.* Social conditions and self-management are more powerful determinants of health than access to care. *Annals of internal medicine* 1998;**129**:406–11. doi:10.7326/0003-4819-129-5-199809010-00011

30 D F. The medical text: between biomedicine and hegemony. *Social science & medicine (1982)* 2004;**59**:1275–85. doi:10.1016/J.SOCSCIMED.2004.01.003

31 Barmania S, Reiss MJ. Health promotion perspectives on the COVID-19 pandemic: The importance of religion. *Global Health Promotion* 2021;**28**:15–22. doi:10.1177/1757975920972992

32 Raven J, Baral S, Wurie H, *et al.* What adaptation to research is needed following crises: A comparative, qualitative study of the health workforce in Sierra Leone and Nepal. *Health Research Policy and Systems* 2018;**16**:1–11. doi:10.1186/s12961-018-0285-1

33 Ali SN, Hanif W, Patel K, *et al.* Ramadan and COVID-19 vaccine hesitancy—a call for action. The Lancet. 2021;**397**:1443–4. doi:10.1016/S0140-6736(21)00779-0

34 Razai MS, Kankam HKN, Majeed A, *et al.* Mitigating ethnic disparities in covid-19 and beyond. *The BMJ* 2021;**372**. doi:10.1136/bmj.m4921

35 Marshall K, Smith S. Religion and Ebola: Learning from experience. The Lancet. 2015;**386**:e24–5. doi:10.1016/S0140-6736(15)61082-0

36 Yezli S, Khan A. COVID-19 social distancing in the Kingdom of Saudi Arabia: Bold measures in the face of political, economic, social and religious challenges. *Travel Medicine and Infectious Disease* 2020;**37**. doi:10.1016/j.tmaid.2020.101692

37 Practical considerations and recommendations for religious leaders and faith-based communities in the context of COVID-19 Interim guidance 7 April 2020 Background. http://data.unaids.org/pub/report/2010/jc1786_fbo_en.pdf (accessed 9 Jun 2021).

38 Hahn RA, Truman BI. Education improves public health and promotes health equity. *International Journal of Health Services* 2015;**45**:657–78. doi:10.1177/0020731415585986

39 Himmelstein DU, Woolhandler S. Health Insurance Status and Risk Factors for Poor Outcomes With COVID-19 Among U.S. Health Care Workers: A Cross-Sectional Study. Annals of internal medicine. 2020;**173**:410–2. doi:10.7326/M20-1874

40 Lazarus J v., Wyka K, Rauh L, *et al.* Hesitant or Not? The Association of Age, Gender, and Education with Potential Acceptance of a COVID-19 Vaccine: A Country-level Analysis. *Journal of Health Communication* 2020;**25**:799–807. doi:10.1080/10810730.2020.1868630

41 Mbuagbaw L, Aves T, Shea B, *et al.* Considerations and guidance in designing equity-relevant clinical trials. BioMed Central Ltd. 2017. doi:10.1186/s12939-017-0591-1

42 Link BG, Phelan JO. Social Conditions as Fundamental Causes of Disease*. *Journal of Health and Social Behavior* 1995;:80–94.

43 Darin-Mattsson A, Fors S, Kåreholt I. Different indicators of socioeconomic status and their relative importance as determinants of health in old age. *International Journal for Equity in Health* 2017;**16**:1–11. doi:10.1186/s12939-017-0670-3

44 Duncan GJ, Daly MC, McDonough P, *et al.* Optimal indicators of socioeconomic status for health research. *American Journal of Public Health* 2002;**92**:1151–7. doi:10.2105/AJPH.92.7.1151

45 Adler NE, Newman K. Socioeconomic disparities in health: Pathways and policies. *Health Affairs* 2002;**21**:60–76. doi:10.1377/hlthaff.21.2.60

46 Evans MK. Health Equity — Are We Finally on the Edge of a New Frontier? *New England Journal of Medicine* 2020;**383**:997–9. doi:10.1056/nejmp2005944

47 Kavanagh NM, Goel RR, Venkataramani AS. County-Level Socioeconomic and Political Predictors of Distancing for COVID-19. *American Journal of Preventive Medicine* 2021;**61**:13–9. doi:10.1016/j.amepre.2021.01.040

48 Jutz R. The role of income inequality and social policies on income-related health inequalities in Europe. *International Journal for Equity in Health* 2015;**14**:1–14. doi:10.1186/s12939-015-0247-y

49 Villalonga-Olives E, Kawachi I. The dark side of social capital: A systematic review of the negative health effects of social capital. *Social Science and Medicine* 2017;**194**:105–27. doi:10.1016/j.socscimed.2017.10.020

50 Reporting guidelines under development for observational studies | The EQUATOR Network. https://www.equator-network.org/library/reporting-guidelines-under-development/reporting-guidelines-under-development-for-observational-studies/#STROBEeq (accessed 9 Jun 2021).

51 Bambra C, Riordan R, Ford J, *et al.* The COVID-19 pandemic and health inequalities. Journal of Epidemiology and Community Health. 2020;**74**:964–8. doi:10.1136/jech-2020-214401

52 Dunne EM, Morgan E, Wells-Moore B, *et al.* COVID-19 Outbreaks in Correctional Facilities with Work-Release Programs — Idaho, July–November 2020. *MMWR Morbidity and Mortality Weekly Report* 2021;**70**:589–94. doi:10.15585/mmwr.mm7016a3

53 Uphoff EP, Pickett KE, Cabieses B, *et al.* A systematic review of the relationships between social capital and socioeconomic inequalities in health: a contribution to understanding the psychosocial pathway of health inequalities. *International Journal for Equity in Health 2013 12:1* 2013;**12**:1–12. doi:10.1186/1475-9276-12-54

54 Imbulana Arachchi J, Managi S. The role of social capital in COVID-19 deaths. *BMC Public Health* 2021;**21**:1–9. doi:10.1186/s12889-021-10475-8

55 Garber K, Fox C, Abdalla M, *et al.* Estimating access to health care in Yemen, a complex humanitarian emergency setting: a descriptive applied geospatial analysis. *The Lancet Global Health* 2020;**8**:e1435–43. doi:10.1016/S2214-109X(20)30359-4

56 Zard M, Lau LS, Bowser DM, *et al.* Leave no one behind: ensuring access to COVID-19 vaccines for refugee and displaced populations. Nature Medicine. 2021;**27**:747–9. doi:10.1038/s41591-021-01328-3

57 Allan B SJ. First Peoples, second class treatment: The role of racism in the health and well-being of Indigenous peoples in Canada. Toronto: 2015. www.wellesleyinstitute.com. (accessed 7 Jun 2021).
58 Shakespeare T, Ndagire F, Seketi QE. Triple jeopardy: disabled people and the COVID-19 pandemic. The Lancet. 2021;**397**:1331–3. doi:10.1016/S0140-6736(21)00625-5
59 Race I. COVID-19 Infections in Manitoba: Race, Ethnicity, and Indigeneity - External report. 2021. https://www.gov.mb.ca/health/cppho/docs/ps/health_equity.pdf (accessed 9 Jun 2021).
60 Epistemonikos Foundation. Methods for the special L.OVE of Coronavirus infection (COVID-19). https://app.iloveevidence.com/covid19 (accessed 14 Jun 2021).
61 Driedger SM, Cooper E, Jardine C, *et al.* Communicating Risk to Aboriginal Peoples: First Nations and Metis Responses to H1N1 Risk Messages. *PLoS ONE* 2013;**8**:e71106. doi:10.1371/journal.pone.0071106
62 Driedger SM, Maier R, Furgal C, *et al.* Factors influencing H1N1 vaccine behavior among Manitoba Metis in Canada: A qualitative study. *BMC Public Health* 2015;**15**:1–15. doi:10.1186/s12889-015-1482-2
63 Mosby I, Swidrovich J. Medical experimentation and the roots of COVID-19 vaccine hesitancy among Indigenous Peoples in Canada. CMAJ. 2021;**193**:E381–3. doi:10.1503/cmaj.210112
64 Indigenous community research partnerships can help address health inequities. https://theconversation.com/indigenous-community-research-partnerships-can-help-address-health-inequities-152705 (accessed 9 Jun 2021).
65 Castellano MB, Reading J. Policy writing as dialogue: Drafting an aboriginal chapter for Canada's tri-council policy statement: Ethical conduct for research involving humans. *International Indigenous Policy Journal* 2010;**1**. doi:10.18584/iipj.2010.1.2.1
66 Tri-Council Policy Statement: Ethical Conduct for Research Involving Humans – TCPS 2 (2018) – Chapter 9: Research Involving the First Nations, Inuit and Métis Peoples of Canada. https://ethics.gc.ca/eng/tcps2-eptc2_2018_chapter9-chapitre9.html?wbdisable=true (accessed 8 Jun 2021).
67 Welch VA, Norheim OF, Jull J, *et al.* CONSORT-Equity 2017 extension and elaboration for better reporting of health equity in randomised trials. In: *BMJ (Clinical research ed.)*. BMJ 2017. j5085. doi:10.1136/bmj.j5085
68 Mallard A, Pesantes MA, Zavaleta-Cortijo C, *et al.* An urgent call to collect data related to COVID-19 and Indigenous populations globally. BMJ Global Health. 2021;**6**:4655. doi:10.1136/bmjgh-2020-004655
69 Calderón-Larrañaga A, Vetrano DL, Ferrucci L, *et al.* Multimorbidity and functional impairment–bidirectional interplay, synergistic effects and common pathways. Journal of Internal Medicine. 2019;**285**:255–71. doi:10.1111/joim.12843
70 Abebe F, Schneider M, Asrat B, *et al.* Multimorbidity of chronic non-communicable diseases in low- and middle-income countries: A scoping review. *Journal of Comorbidity* 2020;**10**:2235042X2096191. doi:10.1177/2235042x20961919

71 Fathi M, Vakili K, Sayehmiri F, *et al.* The prognostic value of comorbidity for the severity of COVID-19: A systematic review and meta-analysis study. *PLoS ONE* 2021;**16**. doi:10.1371/journal.pone.0246190

72 Krieger N, Fee E. Man-made medicine and women's health: The biopolitics of sex/gender and race/ethnicity. International Journal of Health Services. 1994;**24**:265–83. doi:10.2190/LWLH-NMCJ-UACL-U80Y

73 Geldsetzer P, Reinmuth M, Ouma PO, *et al.* Mapping physical access to health care for older adults in sub-Saharan Africa and implications for the COVID-19 response: a cross-sectional analysis. *The Lancet Healthy Longevity* 2020;**1**:e32–42. doi:10.1016/S2666-7568(20)30010-6

74 Mallipattu SK, Jawa R, Moffitt R, *et al.* Geospatial distribution and predictors of mortality in hospitalized patients with COVID-19: A cohort study. *Open Forum Infectious Diseases* 2020;**7**. doi:10.1093/ofid/ofaa436

75 Gaglioti AH, Li C, Douglas MD, *et al.* Population-Level Disparities in COVID-19: Measuring the Independent Association of the Proportion of Black Population on COVID-19 Cases and Deaths in US Counties. *Journal of Public Health Management and Practice* 2021;**27**:268–77. doi:10.1097/PHH.0000000000001354

76 Wiley Z, Ross-Driscoll K, Wang Z, *et al.* Racial and Ethnic Differences and Clinical Outcomes of COVID-19 Patients Presenting to the Emergency Department. *Clinical Infectious Diseases* Published Online First: 2 April 2021. doi:10.1093/cid/ciab290

77 Nazroo J, Becares L. Evidence for ethnic inequalities in mortality related to COVID-19 infections: Findings from an ecological analysis of England. *BMJ Open* 2020;**10**:41750. doi:10.1136/bmjopen-2020-041750

78 Abdalla S, Katz EG, Darmstadt GL. Gender and the impact of COVID-19 on demand for and access to health care: analysis of data from Kenya, Nigeria, and South Africa. *The Lancet Global Health* 2021;**9**:S7. doi:10.1016/s2214-109x(21)00115-7

79 Hur K, Price CPE, Gray EL, *et al.* Factors Associated With Intubation and Prolonged Intubation in Hospitalized Patients With COVID-19. *Otolaryngology - Head and Neck Surgery (United States)* 2020;**163**:170–8. doi:10.1177/0194599820929640

80 Edwards B, Biddle N, Gray M, *et al.* COVID-19 vaccine hesitancy and resistance: Correlates in a nationally representative longitudinal survey of the Australian population. *PLoS ONE* 2021;**16**:e0248892. doi:10.1371/journal.pone.0248892

81 Amorim Filho LI, Landmann Szwarcwald CI, de Oliveira Garcia Mateos SI, *et al.* Seroprevalence of anti-SARS-CoV-2 among blood donors in Rio de Janeiro, Brazil , Josiane Iole França Lopes III , Luis Cristovão de Moraes Sobrino Porto VII , Alexandre Chieppe VIII , Guilherme Loureiro Werneck IV,IX , Grupo Hemorio de Pesquisa em Covid-19. doi:10.11606/s1518-8787.2020054002643

82 Dodd RH, Dadaczynski K, Okan O, *et al.* Psychological wellbeing and academic experience of university students in australia during covid-19. *International Journal of Environmental Research and Public Health* 2021;**18**:1–12. doi:10.3390/ijerph18030866

83 Sosa-Rubí SG, Seiglie JA, Chivardi C, *et al.* Incremental risk of developing severe covid-19 among mexican patients with diabetes attributed to social and health care access disadvantages. *Diabetes Care* 2021;**44**:373–80. doi:10.2337/dc20-2192

84 Romer D, Jamieson KH. Conspiracy theories as barriers to controlling the spread of COVID-19 in the U.S. *Social Science and Medicine* 2020;**263**. doi:10.1016/j.socscimed.2020.113356

85 García-Portilla P, de la Fuente Tomás L, Bobes-Bascarán T, *et al.* Are older adults also at higher psychological risk from COVID-19? *Aging and Mental Health* Published Online First: 2020. doi:10.1080/13607863.2020.1805723

86 Welch V, Tugwell P, Petticrew M, *et al.* How effects on health equity are assessed in systematic reviews of interventions. *Cochrane Database of Systematic Reviews* Published Online First: 8 December 2010. doi:10.1002/14651858.mr000028.pub2
